# Barriers and facilitators to the implementation of palliative care services at five tertiary hospitals in Nigeria: a qualitative formative study

**DOI:** 10.1186/s12913-025-13138-1

**Published:** 2025-07-23

**Authors:** Chisom Obiezu-Umeh, Ann Abiola Ogbenna, Lyra S. Johnson, Matthew T. Caputo, Lisa R. Hirschhorn, Tonia Chinyelu Onyeka, Akinwale Mukaila Oyegbade, Geraldine Ugochinyere Ndukwu, Israel Kayode Kolawole, Ayilara Saheed Alao, Gracia Ker Eke, Nadia Adjoa Sam-Agudu, Ashti Doobay-Persaud

**Affiliations:** 1 Department of Medical Social Sciences, Northwestern University Feinberg School of Medicine, Chicago, IL USA; 2 Department of Haematology and Blood Transfusion, College of Medicine, University of Lagos, Lagos, Nigeria; 3 Department of Haematology and Blood Transfusion, Lagos University Teaching Hospital, Lagos, Nigeria; 4 Center for Global Health Education, Robert J. Havey, Institute for Global Health, Northwestern University Feinberg School of Medicine, Chicago, MD, Illinois USA; 5 Ryan Family Center for Global Primary Care, Robert J. Havey, MD Institute for Global Health, Feinberg School of Medicine, Northwestern University, Chicago, IL USA; 6 Department of Anaesthesia/Pain & Palliative Care Unit, College of Medicine, University of Nigeria, Enugu, Nigeria; 7 IVAN Research Institute, Enugu, Nigeria; 8 Hospice & Palliative Care Department, College of Medicine, University of Ibadan, Ibadan, Nigeria; 9 Department of Family Medicine, University of Port-Harcourt Teaching Hospital, Port-Harcourt, Rivers State Nigeria; 10 Department of Anaesthesia, University of Ilorin, Ilorin, Nigeria; 11 Department of Pain and Palliative Medicine, Federal Medical Center, Abeokuta, Ogun State Nigeria; 12 Department of Paediatrics, University of Port-Harcourt Teaching Hospital, Port- Harcourt, Rivers State Nigeria; 13 International Research Center of Excellence, Institute of Human Virology Nigeria, Abuja, Nigeria; 14 Global Pediatrics Program and Division of Pediatric Infectious Diseases, Department of Pediatrics, University of Minnesota Medical School, Minneapolis, USA; 15 Department of Pediatrics and Child Health, School of Medical Sciences, University of Cape Coast, Cape Coast, Ghana; 16 Department of Medicine, Division of Hospital Medicine, Northwestern University Feinberg School of Medicine, Chicago, IL USA

**Keywords:** Palliative care, Qualitative content analysis, Implementation science, Consolidated framework for implementation research, Nigeria

## Abstract

**Background:**

Palliative care (PC) is critical for improving the quality of life for individuals with life-limiting illnesses, yet it remains inaccessible or underdeveloped in low- and middle-income countries like Nigeria. Understanding the factors influencing PC implementation is a crucial first step toward strengthening health system readiness and responsiveness to meet the growing demand for PC services in Nigeria and similar settings.

**Methods:**

We conducted five in-depth interviews and ten focus group discussions to explore health provider, patient and caregiver perspectives on barriers and facilitators to PC implementation at five geographically diverse PC units in Nigeria. Guided by the Consolidated Framework for Implementation Research (CFIR), qualitative data were analyzed using the framework approach.

**Results:**

Barriers and facilitators spanned all five CFIR domains (Innovation, Outer Setting, Inner Setting, Individuals, and Implementation Process). Barriers to PC implementation included, (1) patient financial constraints, (2) inadequate facility funding and resources, (3) shortage of PC providers, 4) delayed/late referrals, 5) Inaccurate knowledge and negative beliefs about PC among health providers and patients, and (6) substandard opioid medications. Facilitators included, (1) high value for PC among patients and caregivers, (2) positive attitudes and (3) desire for PC knowledge and training among PC providers, (4) strong provider motivation and commitment to improve patient quality of life.

**Conclusions:**

Our study identified key multi-level factors influencing PC implementation at PC centers in Nigeria. These findings will inform the expansion and integration of PC services into local health systems across Nigeria.

**Supplementary Information:**

The online version contains supplementary material available at 10.1186/s12913-025-13138-1.

## Background

Palliative care (PC) is defined as an active, total care approach to addressing the psychological, physical, social, and spiritual needs of individuals with life-limiting illnesses and their families to facilitate coping and improve quality of life, and it is increasingly recognized as a global priority [1–4]. While PC has historically focused on patients with cancer, recent evidence suggests greater benefits for individuals with other chronic illnesses—such as heart failure, kidney disease, and dementia—including those in the earlier stages of illness [1]. Despite being acknowledged as an essential component of care [5], PC remains inaccessible to many people, particularly in low- and middle-income countries (LMICs) [6]. It is estimated that of the 57 million people worldwide who could benefit from PC annually, 76% reside in LMICs, where the unmet needs for PC are particularly high [4, 7, 8]. This disparity between demand and supply can be attributed to the limited availability and accessibility of PC services, which in many LMICs are either non-existent, fragmented, or operate outside the national health systems [9, 10].

In Nigeria, the most populous country in Africa, only 17 PC facilities—mostly concentrated and siloed within tertiary public hospitals—serve a population of over 200 million [9]. With increased government attention, promotion of PC, and rising prevalence of cancer and other chronic illnesses, the demand for PC has been increasing in Nigeria [9]. However, the health system’s readiness and response remain inadequate to meet this increased need [11]. Furthermore, existing care models, which are predominantly hospital-based, are suboptimal due to various social and systemic barriers, including inadequate healthcare infrastructure and resources, shortage of PC specialists, and stigma [12–15]. These challenges underscore the urgent need for evidence-based strategies to improve the availability of and access to timely, high-quality PC services [16, 17].

Implementation science offers the potential to advance current PC practices and inform future efforts to promote scale-up and consistent use of PC services across diverse clinical settings and healthcare systems [1, 18]. Learning from real-world case experiences provides valuable insights into which PC practices work, for whom, how to implement them and under what context [19]. Identifying factors (i.e., determinants) that influence PC implementation is a critical first step in generating generalizable insights for future research and policy development in Nigeria, where evidence in this area remains relatively limited. The Consolidated Framework for Implementation Research (CFIR) provides a robust theoretical framework to systematically explore and understand these determinants across five key domains [20, 21]. Guided by CFIR, the primary aim of this study is to examine the barriers to and facilitators of implementing PC services from the perspective of health providers, patients and caregivers, at tertiary public hospitals in Nigeria. Findings from this study will inform a planned study to evaluate the evidence-based strategies to implement palliate care in tertiary hospital settings among patients and their families.

## Methods

### Study design

We conducted semi-structured, in-depth interviews (IDI) and focus group discussions (FGDs) from February to April 2023. The Consolidated Criteria for Reporting Qualitative Research (COREQ) [22] guided data analysis and reporting.

### Study setting and participants

Sites A, B, C, D, and E (anonymized) are PC units located within tertiary hospitals across four of Nigeria’s six geopolitical zones: North-Central (*n* = 1), South-West (*n* = 2), South-East (*n* = 1), and South-South (*n* = 1). These sites were purposefully selected from an existing US-Nigeria consortium of interprofessional providers dedicated to advancing the education, training and research on PC in Nigeria. Study participants included PC providers (physicians and nurses) as well as patients and their caregivers, from the five PC units. The head of each PC unit, who was also a member of the consortium, was asked to identify providers involved in patient care within the unit for at least one year and who were knowledgeable about its operations. These providers were subsequently invited to participate. The participating PC providers were then asked to enlist patients and their caregivers who were actively receiving PC services at the time of the study, were 18 years or older. From this list, patients and caregivers were randomly selected and invited to participate in the study either by phone or during a routine clinic visit, with up to four patient-caregiver dyads recruited from each unit.

### Procedure

Focus group discussions were conducted among patients, and in-depth interviews were conducted among the PC providers. Semi-structured, open-ended interview guides were developed for each constituent group based on the five domains of CFIR (innovation characteristics, inner setting, outer setting, characteristics of individuals, and implementation process) (see Additional file 1 and 2). PC provider interviews focused on determinants of current PC implementation, while interviews with the patients and caregivers focused on their understanding of PC and experiences as PC recipients. In-person interviews and FGDs were conducted by two trained female study team members with extensive experience in conducting qualitative interviews and lasted approximately 45 to 60 min. Interview sessions were conducted in English, Yoruba or Igbo (native languages of participants) and audio recorded using a digital recording device. Prior to the start of each interview, participants were briefed on the study objectives, potential risks, and benefits. Written informed consent was sought and received from all participants. After each interview, audio-recorded files were transcribed verbatim, translated into English (where needed) and reviewed for accuracy.

### Data analysis

We applied a framework approach to analyze the data using Dedoose software [23]. This qualitative data analysis approach has five key steps: data familiarization, the iterative development of a thematic framework, indexing data to categorize it within the framework, summarizing the data within each category, and interpreting the data [24]. The coding framework was structured around the five CFIR domains. Although the original CFIR 1.0 informed the development of the interview guides, we consulted both the original and updated CFIR 2.0 version for the analysis. At the outset, two researchers (CO and LJ) selected and independently double-coded four transcripts using priori CFIR codes while also creating new codes for emergent themes. Both researchers met routinely to align definitions and interpretations of existing codes, compare their coding, discuss emergent themes, and resolve any discrepancies. One researcher (CO) subsequently coded the remaining transcripts using the established coding framework. The data were further categorized into barriers and facilitators, summarized, and compared across constituent groups and PC units, with careful attention to identifying patterns and unique statements. To ensure the credibility (internal validity) of the findings, the preliminary data summary was presented to a few participants to confirm that their views were accurately reflected.

## Results

In total, five PC providers participated in in-depth interviews, including two physicians and three nurses, and 29 patients and 29 caregivers participated in ten focus group discussions. The demographic characteristics of the participants are displayed in Table 1.

**Table 1:** Demographic characteristics of the participants

Characteristic	No. of participants		
	**Physicians/Nurses (*n*** **= 5)**	**Patients (*n*** **= 29)**	**Caregivers** (***n*** **= 29)**
Age in years, median (IQR)	50.0 (48.0, 56.0)	44.0 (36.0, 63.0)	48.0 (33.8, 56.8)
Sex, n (%)			
Female	5 (100.0)	19 (65.5)	18 (62.1)
Male	0 (0.0)	10 (34.5)	10 (34.5)

Some frequencies do not add up to the total due to missing observations

*Abbreviations:IQR* Interquartile range

Table 2 summarizes the barriers and facilitators to implementing palliative care services, categorized into the five CFIR domains: innovation characteristics, inner setting, outer setting, characteristics of individuals, and implementation process. Additional quotes are provided in Additional file 3.

**Table 2:** Barriers and facilitators of implementing palliative care services according to CFIR domains

Domain	Theme	Barrier (-), Facilitator (+), or Both (+/-)
Innovation	Perceived benefits of PC	+
Inner setting	Informal funding arrangements	+
	Resources and funding support	-
	Organizational structure	-
	Delayed referral	-
Outer setting	Traditional care	-
	Quality control of medication	-
Characteristics of Individuals	Knowledge of and beliefs about palliative care	-
	Attitudes towards palliative care among non-PC providers	-
	Patients and caregivers’ experiences with non-PC providers	-
	PC provider attitudes	+
	Financial constraints	-
Process	Systematic PC education & training	+/-

*Abbreviations*: *PC* palliative care

### Innovation

#### Perceived benefits of PC

PC was perceived to have a significant impact on patients’ and caregivers’ psychological and physical health. Patients described how their physical health had considerably improved, especially helping with pain relief and symptom control.

*“The first time we got here*,* I was on admission then*,* I couldn’t sleep. Then I knew them [palliative care] and they really helped me because the pain was very severe then. So*,* when they came to check up on me*,* they gave me another victory*,* a different medicine from the one I was using before*,* it went well with my body*,* and the pain left. I can say the pain was rated as 9 as of then and they introduced me [to palliative care]*,* it dropped to 5*.” (Patient, Site A).

Several patients strongly expressed that they experienced psychological improvement as a result of the care and support received from the team, describing feeling encouraged, hopeful, and better equipped to cope with and manage their condition.

*“When I was now on admission during my surgery I met with this department [palliative]. She came with some other staff too… They have been talking to me*,* encouraging me. Maybe we are down or restless*,* and somebody encourages you and you have hope again*,* your hope comes alive again*,* and you have gotten support.”* (Patient, Site D).

Similarly, a salient aspect of PC expressed by many patients and caregivers was a deep sense of spirituality and how the spiritual counseling aspect of PC services was also a source of courage, strength, comfort, and normalcy.

*“Palliative care is by divine*,* by divine…I am very happy for their prayer. The prayer they prayed for me that day built up my faith. And that’s how I summoned courage.*” (Patient, Site D).

While patients’ and caregivers’ accounts mostly focused on the highly valued psychological support and pain management received, they also noted the significance of the care team going above and beyond to cater to their financial needs, including medical expenses and nutritional support.

*“They will ask my sister*,* “Have you eaten?” If you don’t have money and there is nobody you can call for any assistance*,* they are ready to give us the assistance*.” (Caregiver, Site C).

Patients noted that they benefited from follow-up care, as well as reminders for medications and subsequent medical visits, provided by the palliative care unit following their discharge. While discussing the difficulties of providing home-based care services for all their patients due to logistical issues, a few PC providers highlighted the benefits of offering some form of bereavement follow-up services to the affected families.

*“We do visitations*,* we do home care also and we do bereavement care after the patient has passed. You know we have been going there to see the patient*,* now that the patient is no more*,* we want to see how the family is coping*,* if there’s anything they need*,* any solution*,* especially those that like especially have issues* (Nurse, Site A).

### Inner setting

#### Informal funding arrangements

Many patients admitted to the PC unit faced significant financial constraints, which often limited their ability to consistently access PC services and afford essential medications. In recognition of this, the PC units depended heavily on charitable funding and donations. PC providers frequently contributed their own funds to help cover the medical expenses of patients in need, largely driven by a shared commitment to improving patient quality of life and a strong sense of community within the unit.

*“If they can’t afford it*,* we’ll have mercy*,* we’ll have mercy because most of those patients*,* they’ve spent a lot*,* they’re drained already.”* (Nurse, Site A).

*“We contribute. All of us contribute money to assist them. We contribute money. Sometimes I open the WhatsApp group*,* asking for donations. Very tiny*,* just getting tiny donations.”* (Physician, Site D).

In addition, one facility implemented a sliding scale payment model, allowing patients to be billed based on their financial capacity.

*“When you are seeing us personally*,* our main aim is not finance*,* because most of the time the patient may come in distress and in pain. So*,* if the patient has money*,* we bill the patient for about 3*,*000 Naira. Then subsequent visits 1*,*000 Naira based on their financial state. If the patient does not have money*,* the patient can pay the money another time.” (Physician*,* Site B)*.

####  Resources and funding support

Resource deficiencies were frequently highlighted by PC providers as a significant barrier to the provision of PC. Several providers also reported a lack of funding support from management, as the PC unit was perceived as generating little economic benefit for the hospital system. This perception in turn contributed to the limited financial resources allocated to the unit, further impacting their ability to provide PC services.

*“Because it is like the last stage of medicine*,* it’s like the last element of medicine so sometimes we are neglected. So*,* by the time they distribute some things within the hospital*,* they will think of palliative care last and we will not get whatever has actually been distributed to help in support of the patients”* (Physician, Site B).

*“The hospital doesn’t see palliative care as that. See the hospital even feels we don’t generate funds. They only allow us because they feel we are somehow in oncology. You know*,* that’s all. But they don’t feel we are actually*,* as far as we are not generating funds.”* (Physician, Site D).

Most PC providers cited a shortage of specialists and the understaffed nature of palliative care units as significant challenges. Quite a number of the PC providers were not full-time; they divided their time between multiple units and took on other hospital assignments, resulting in frequent understaffing, work overload, and high turnover rates. Additionally, the high turnover rates were exacerbated by the migration of healthcare workers outside Nigeria due to the brain drain crisis, commonly referred to as “japa syndrome” in the country.

*“The Japa syndrome has affected us*,* three of our nurses have gone and it’s only me that is left now*,* so we’re still looking for nurses. nobody to relieve me*,* that okay go and support her*,* I’ll say go to the ward and see*,* everywhere is short staffed. But when this japa syndrome started*,* since 2020 most of the people that we have trained*,* have japa. So*,* we have new hands-on ground who doesn’t even know or have what it takes to be a palliative or how to nurse a patient.”* (Nurse, Site A).

####  Organizational structure

Providers reported a lack of formalized structures dedicated specifically to PC services as a barrier to accessibility. They described how palliative care units were often embedded within other departments, such as oncology, and typically limited to treatment rooms or small private spaces.

*“The only thing management has given us is this room that has been there initially and this space*,* that’s all. No unit. We are just on our own.”* (Physician, Site D).

####  Delayed referral

Regarding decisions about when patients should be referred to for PC, providers reported a limited understanding and a lack of clear referral criteria, which often resulted in delays. Both providers and patients noted that non-PC providers typically initiated referrals at late stages of the disease, when symptoms were more pronounced or after curative options had been exhausted.

*“That is one of the challenges that we are seriously facing*,* the consult is the issue*,* because most of them when they see the impact of palliative care on their patient*,* they believe the palliative care would sustain their patient*,* and because of that*,* they hold on for most of them and most of the times that they’re supposed to release the patient to receive the best care*,* they hold on to the patient until when the patient is at the later end.”* (Nurse, Site A).

### Outer setting

#### Traditional care

Some patients reported resorting to traditional remedies after their diagnosis or during treatment, either because they could not afford ongoing medication or in hopes of finding a cure. This often led to inadequate treatment and delays in seeking palliative care, which could worsen their condition.

*“We started treating the splenomegaly the native way. Even to the extent that they made me step on the stomach. So*,* before you know it*,* instead of it to go*,* things started increasing gradually gradually*,* and the boy became so weak that even the flesh you can’t see the flesh. He is very lean… people with this critical illness are not financially buoyant to take care of it. And by so doing they find a way to get alternative medication and before you know it the thing will give them up and they die.”* (Caregiver, Site D).

#### Quality control of medications

Several providers expressed concerns regarding the quality and effectiveness of pain relief medications available across the country, specifically highlighting issues related to the potential dilution of drugs and the prevalence of counterfeit opioid analgesics. They emphasized the urgent need for high-quality pain relief options to ensure effective treatment for patients.

*“The quality of drug*,* pain relief drug is an issue*,* take for instance morphine*,* is the most pain relief drug that they use here. But in my sister’s case*,* … we complained. You’ll find out that after giving you*,* or your patient morphine*,* the pain will still be there… So yesterday we complained*,* and they were like maybe it’s the way they dilute … you know even if all other drugs are inferior let pain drugs be original.”* (Caregiver, Site E).

### Characteristics of individuals

#### Knowledge of and beliefs about palliative care

Inaccurate knowledge and negative beliefs toward PC among non-PC healthcare providers included equating PC to giving up or end-of-life care; these were identified as significant barriers that limited PC uptake early in patients’ illness trajectory. Similarly, most patients and caregivers reported initial hesitancy towards receiving PC due to lack of knowledge and understanding, associating such services with negative connotations of death, loss of hope for finding a cure or treatment, and managing terminal illnesses.

*“[A caregiver] was saying they told her palliative would be coming to see her sister*,* she just remembered that palliative is just something [about]managing*,* managing. It means her condition cannot be resolved so they’re going to be managing it until she dies. That is the impression because she has heard*,* you know the Nigerian factor*,* they’ll say go and palliate that road*,* go and palliate this place*,* go and palliate*,* they know it’s not the lasting solution*,* but you’ll just manage. They all know palliative as managing. So*,* if the patient is someone who has heard that word before*,* they will relate it to that situation and say*,* it means this thing there’s no lasting solution to this*,* it means you’re going to be managed…and it really affects them.”* (Nurse, Site A).

This perception prompted a PC provider to question whether “palliative care” is the most suitable term to describe the services offered by the PC unit. One provider explained that *“Immediately when patients hear “palliative*,*” they think we are sharing rice and beans”* (Physician, Site D), referring to the government’s palliative program during the peak of the COVID-19 pandemic, which focused on addressing social welfare needs of Nigerians.

#### Attitudes towards palliative care among non-PC providers

Several PC providers described issues concerning collaboration and coordination with non-palliative or referring providers, noting that PC services were perceived as interfering with patient care or overstepping boundaries. As a result, there was a lack of acceptance of such services among non-palliative health providers, contributing to delays in patients’ referrals, and reluctance to request PC consultations for patients who might have benefited from such services earlier.

*“The co-worker would have even*,* poisoned the minds of the patients and the relation*,* so for them to come here [PC unit] would be a problem. We will see some doctors*,* [when a] patient needs palliative care. In fact*,* they will say “palliative care*,* that’s the end. If the patient has not gotten to the end stage*,* don’t send them to palliative”. You will see a consultant who will be telling me that I want to trap his patient*.” (Nurse, Site A).

#### Patient and caregiver experiences with non-PC providers

Prior negative experiences with providers were reported to have influenced patients’ attitudes toward initiating PC. Some patients and their caregivers felt that they received minimal support from their primary care team following diagnosis of illness.

*“There are differences between the ones here and other nurses or other staffs [in the hospital]*,* that when you are in the ward*,* they treat you like a nobody*,* they will be shouting at you like you are a nobody*,* in fact they will neglect you even if you are dying.”* (Patient, Site C).

#### Palliative care provider attitudes

Majority of patients and their caregivers described the quality of care received from PC providers as exceptional, stating that they felt “loved” by the staff and experienced a more responsive care environment, which was unprecedented for many. Characteristics used to describe PC providers included “loving”, “caring”, “compassionate”, “dedicated”, “encouraging”, “kind”, “friendly”, and attentive listeners who cater to their needs.

*“I’ve been through a lot but when I got to their [PC]unit*,* they don’t only deal with medicine there. They do not only prescribe medicine. Their doctors will come*,* their nurses will come*,* they will give you comfort words*,* that you won’t feel less loved*,* they will converse with you in a lovely and comforting manner*,* they will explain to you if you feel any pain…so they are really trying amongst every other unit in the [hospital]*,* I will like to say they are still the best.”* (Patient, Site A).

*“For the few days and weeks*,* we have been in this unit called palliative care*,* we have seen number one the love. Number two*,* I have seen their care*,* prayers and very importantly their huge smiles on our lives. At least that quality goes a long way.”* (Caregiver, Site D).

Notably, most patients and their caregivers reported a significant change in their initial perceptions of PC after being admitted to the unit. There was a more positive attitude, a shift they attributed to interactions with PC staff and the quality of care received by their providers. A few patients expressed that the staff in the PC unit were more accessible than those in other departments, and they benefited from the multi-disciplinary team approach to care.

*“This department is loved*,* because they come in [as a] team*,* they’re so caring*,* because the first day they came*,* there were five individuals*,* after that the following Monday when they came*,* they are not less than ten. I am surprised about the pamper[ing] and care…they are one of the best departments*.” (Patient, Site A).

#### Financial constraints

Most patients and caregivers expressed financial constraints that often limited their ability to access PC services and medications. PC providers corroborated this concern, highlighting that many patients admitted to palliative care faced difficulties in meeting basic needs and affording treatment due to their inability to work because of their illness, further exacerbated by the prevailing national economic conditions.

*“For patients most of them they cannot afford the narcotic drug most especially when they are in excruciating pain*,* and we have drugs for them to go and for sure some of them they cannot afford it*,* so the financial constraint is there.”* (Nurse, Site C).

### Process

#### Systematic PC education & training

While many PC providers reported some prior training in PC, most highlighted a lack of systematic education and training on these services within the hospital and education systems for other providers. The majority indicated that they received their training or certifications from accredited institutions outside Nigeria. All providers expressed a strong desire to learn more about PC services to enhance patient quality of life. To address this need, they suggested creating robust, PC-focused training programs, including in-service training and booster sessions that could be integrated into onboarding processes or routine departmental meetings to further improve their knowledge and skills.

*“You know palliative training is a problem*,* the fact that in Nigeria presently there’s no place where there’s training or a certificate course training*,* most of the training that we did are short courses…but as it is now*,* we can’t do the training here [in Nigeria]*,* we have to go outside the country.”* (Nurse, Site A).

## Discussion

The qualitative study provides a robust examination of the barriers and facilitators influencing the implementation of palliative care services in tertiary hospitals in Nigeria. As the first known multi-center implementation science study on PC in the country, our findings add to the limited but growing literature on PC implementation in Nigeria, which operate within a unique set of structural constraints and challenges. One key finding was that all constituent groups —providers, patients, and caregivers—identified implementation determinants across ecological domains, signaling the need for multi-level coordination to support the integration of PC and ultimately improve patient outcomes.

Negative perceptions and attitudes towards PC among healthcare providers, such as equating PC to end-of-life care, were identified as significant barriers to timely referrals and adoption by non-PC providers in this study. Similar perspectives have been reported by attending or referring providers in other African countries, including Ghana and Ethiopia [25, 26]. The present study highlights a recurring concern among non-PC providers about PC instilling hopelessness and fear in patients, a trend also documented by other studies in Nigeria, Uganda, Kenya and Malawi [14, 27, 28]. This was further corroborated by our interviews with patients and caregivers, who initially associated PC with death and the loss of hope for a cure. Moreover, our findings reveal that some non-PC providers view PC as interfering with their patient care, potentially explaining why PC initiation is often delayed until all curative options have been exhausted.

To resolve these delays in referral, comprehensive education of healthcare workers and referral providers is needed to reframe PC as a holistic approach that can complement patient management and enhance quality of life at all stages of illness. Misconceptions about PC have been attributed to a lack of systematic education and training of PC across the continent [29]. Currently, in Nigeria, only 3 out of 40 accredited medical schools have successfully integrated PC education into their curricula [30]. Additionally, health provider in our study reported a significant gap in PC training and the unavailability of certificate courses in Nigeria. This highlights the urgent need to incorporate PC education into medical and nursing curricula, and in continuing education for healthcare workers. Expanding these training opportunities to include non-PC providers and addressing their perceptions of PC could also promote greater acceptance among patients and facilitate the integration of PC at earlier stages of care [17, 31].

Positive experiences with PC among patients and caregivers emerged as a major facilitator of PC accessibility and acceptability, as identified within the Innovation domain of CFIR. A major perceived benefit of PC was its significant impact on patients’ and caregivers’ psychological and physical health – a finding consistently reported in other qualitative studies on PC services [9, 32]. Patients appreciated the interdisciplinary team approach and felt supported by the large teams of practitioners providing integrated care. Interdisciplinary approaches to PC have long been recognized as beneficial to providers, patients, and their families [33]. Patients also expressed appreciation for the comprehensive nature of PC, which included support for home-based care as well as bereavement services for family members in some of the PC units.

As identified by PC providers, the absence of formalized organizational structures to house PC units within tertiary hospitals served as a barrier to both the accessibility and acceptability of PC care, limiting the development of essential facilities that could enhance quality of care and improve the overall experience for patients, families, and staff [34, 35]. Similar to a recent study conducted in Ghana [36], we found that PC services were deprioritized and perceived by hospital management as offering limited economic benefit. As a result, PC units faced significant resource shortages, including inadequate funding support. To address these challenges, the PC units relied heavily on charitable donations, with their providers frequently contributing their own funds to cover the medical expenses of patients in need. Integrating PC into broader health financing frameworks and ensuring appropriate resource allocation could enhance the visibility, stability, and sustainability of hospital-based palliative care services [37, 38].

In LMICs such as Nigeria, financial factors compounded by a lack of health insurance and few/no social protection programs can prevent patients from seeking or receiving early treatment [39]. Our study corroborated this, with several patients and caregivers reporting financial constraints due to the harsh economic conditions, which in turn affected their ability to afford basic needs and access PC services and medications. Additionally, some patients and caregivers reported financial hardship due to spending a significant portion of their resources on care or curative options or from being unable to work due to illness.

Furthermore, the lack of adequate supplies, and substandard opioids in the broader context can severely hinder pain management for patients with serious illnesses. Global access to opioids, such as morphine, is not uniform, with only 0.03% of opioids being distributed to LMICs [40]. Over the last 20 years, opioid consumption in Africa has remained insufficient and stagnant [41], with the prevalence of substandard drugs being a major challenge for Nigeria specifically [42, 43]. Several providers expressed concerns about the quality and effectiveness of pain relief medications available across the country, specifically highlighting issues with drug dilution and the prevalence of counterfeit opioid analgesics. These challenges underscore the urgent need for robust advocacy and policy reforms to ensure the consistent availability of high-quality essential medications for PC within publicly-funded healthcare systems [37].

### Strengths and limitations

Our study has several limitations. Although topic saturation was achieved, the sample size of healthcare providers was small and limited to the few practicing PC physicians and nurses. Further research is needed to include other healthcare professionals (i.e. social workers and chaplains) involved in delivering PC, as well as non-PC providers and patients who did not access palliative care. Also, the perspectives of pediatric and adolescent PC recipients and their caregivers were not explored. We aim to address this in future planned studies, including designing ethically sound protocols that include procedures for interviewing minors. Additionally, the findings in our study may be subject to researcher and selection bias. To minimize the impact of this bias, we shared our findings with a subset of study participants to ensure that their views were accurately reflected. Additionally, all interviews and focus group discussions were conducted by facilitators who were independent of the participating institutions and had no prior relationship with the participants—whether providers, patients, or caregivers. Lastly, our study was conducted in five PC units across four geopolitical zones in Nigeria; as such, the findings may not be representative of PC units in other geopolitical zones. Nonetheless, our study is the first, to our knowledge, to use an implementation science framework such as CFIR to understand the determinants of PC delivery and use in Nigeria. Identifying these contextual factors will help inform strategies to address barriers and leverage facilitators to optimize PC implementation in Nigeria.

## Conclusion

This study captures perspectives from individuals across multiple geographically diverse PC units including providers, patients and their caregivers. As the first of its kind in Nigeria, the findings have the potential to address a significant gap in the literature by informing development and adaptations of PC delivery models and shaping local policies. The barriers and facilitators identified in this study may also be relevant to PC services outside of tertiary hospitals, and potentially, to health systems in other African countries and LMICs.

## Supplementary Information

Supplementary Material 1.

Supplementary Material 2.

Supplementary Material 3.

## Data Availability

The interview data generated and analysed during the current study are not publicly available due to privacy considerations but are available from the corresponding author on reasonable request.

## Abbreviations

COREQ: Consolidated criteria for reporting qualitative research.
CFIR: Consolidated Framework for Implementation Research
FGD: Focus group discussion
LMICs: Low- and middle-income countries
PC: Palliative care
