# Supplementary material for: Barriers and facilitators to the implementation of palliative care services at five tertiary hospitals in Nigeria: a qualitative formative study
Source: BMC Health Serv Res. 2025 Jul 23;25:970. doi: 10.1186/s12913-025-13138-1 (PMC12285048; doi:10.1186/s12913-025-13138-1)
Supplement: Supplementary file 1 — Supplementary Material 1. [file 12913_2025_13138_MOESM1_ESM.pdf]

IDI Guide for Focus Group

Title: Assessment of PC services provided, model/operation of PC unit, and viewpoint of the respondent about PC in general and in establishing a PC unit

Purpose: To explore the PC practices, and patient and caregivers' satisfaction with PC services in the centres

Who to interview/Respondents: Patients and caregivers

N.B. Investigator should obtain signed informed consent using the informed consent form before starting the interview and during the interview should probe for explicit answers where there is any form of ambiguity.

RESPONDENT

A. Institution

Institution:

B. Understanding of PC

What do you understand by PC?

Did you know about PC prior to coming to this centre?

How did you get to know about this centre and the services they provide to patients?

What was your reaction when you were referred here?

Do you belief the reason for being referred here is because there no hope/no future for you (or your loved one)?

Why do you think so?

C. Understanding of patient's/loved one's disease state

What do you understand about your (or your loved one's) disease state?

What are the emotions surrounding to the knowledge of your (loved one's) health condition?

Do you believe the hospital and loved ones can provide the care and attention you need? *(Probe their trust in the care given and their approval of the care)*

Do you think it is worth living with this health condition? *(Probe for explanation of their response)*

Do you think it is worth living life to the fullest with this health condition? *(Explain further if the participants do not get the difference between this question and the previous question. Lead them to consider expenses, discomfort, time, etc.)*

Do you think you (or your loved one is) are eligible in decision making as it relates to their health and life at this point? *(Probe for reason for their answer)*

D. Care received

What kind of care are you currently receiving (or providing)?

On a scale of 1 to 10 with 10 being the highest, how much care and relieve could you give yourself (your loved one) without the PC centre's involvement?

On a scale of 1 to 10 with 10 being the highest, how much do you value the care you are receiving?

Has there been any remarkable improvement since you stated receiving (or giving) care? *(Probe for remarkable changes)*

E. Service Satisfaction

Which of the care that you are receiving (providing) is most important to you?

What service(s) not provided at this centre would you appreciate to be provided?

How much relief will it bring to you?

What services rendered do you desire to be improved?

How much relief will it bring to you if these services are improved?

What qualities do you appreciate in your health care providers?

If you had a constructive counsel to give the team in this centre, what would it be?

What do you think would hinder people from using this centre's services? (Probe for ignorance, costs, distance, culture, religion)

Summary

(Summarize the respondent's answers by itemizing/highlighting crucial points)

Conclusion

Is there something else you would like to communicate about this subject of palliative care at this centre?

Appreciation

(Thank the respondent for their time and sincerity during the interview)
