# Supplementary material for: Barriers and facilitators to the implementation of palliative care services at five tertiary hospitals in Nigeria: a qualitative formative study
Source: BMC Health Serv Res. 2025 Jul 23;25:970. doi: 10.1186/s12913-025-13138-1 (PMC12285048; doi:10.1186/s12913-025-13138-1)
Supplement: Supplementary file 2 — Supplementary Material 2. [file 12913_2025_13138_MOESM2_ESM.pdf]

IDI Guide for Key Interview

Title: Assessment of PC services provided, model/operation of PC unit, and viewpoint of the respondent about PC in general and in establishing a PC unit

Purpose: To explore the PC practices, and PC staff attitudes as well as perceptions about PC in the centres

Who to interview/Respondents: PC Providers

N.B. Investigator should obtain signed informed consent using the informed consent form before starting the interview and during the interview should probe for explicit answers where there is any form of ambiguity

RESPONDENT

A. Socio-demographics

Institution:

PC unit:

Initials:

Age:

Sex:

Occupation/ Work cadre/ Designation:

B. Palliative care service provision

How long have you worked in this institution and in this PC centre?

How often do you actively care for terminally ill patients/ those with chronic diseases *(probe for the period in terms of days, weeks, months, or years)*?

C. Training in/Knowledge of Palliative care

Which palliative care training(s) have you attended *(probe for year, location and duration of training, and certification given)*?

What Knowledge from this training do you still apply in your work?

PC CENTRE

D. Staff

What different cadre of PC staff does your centre have and what is each cadre's monthly remuneration *(probe for any special allowance given? If so how often and how much)*?

Are staff solely full-time PC staff or do they double as both PC unit staff and other unit staff *(if staff have other hospital engagement/responsibility, probe for how they share their time/combine work in the different unit? Does this affect their effectiveness)*?

E. Services offered, infrastructure, and model/operation of palliative care in

What services does your centre offer?

What facility/equipment does our centre possess for the administration of services *(Probe for the availability of a domiciled site, number of rooms and their uses, what specific facility/equipment are available. How does all these benefit the patients and the caregivers?)*?

What are your current operations (Have they helped? Can they be improved? How?)

- How often do you have clinics/consultation, ward rounds, and emergencies?

- Is the centre a hospital department or a stand-alone unit in the institution?

- How are patient identifiers/files stored (electronically or in hardcopy or both ways)?

- Does the centre get referrals (from within and outside the institution)? If yes, how often?

- Which department sends in more referrals?

- Is there a demarcation between paediatric and adult care in your centre?

- What is the average number of patients per month?

What infrastructure does the centre has that attracts people to your services/what makes your services different from other centres?

Could you mention some **managerial and staff rules and responsibilities**

How is the **centre funded**?

What **payment models** do patients use, if at all they pay?

- How does the centre accommodate patients with limited resources available for care?

F. Challenges with the establishment of a palliative care centre

What are the major challenges you encountered while working here and how did your centre overcome these barriers *(probe for remuneration, relationship with colleagues, availability of manpower, education of staff, awareness of PC by the public and the institution, sponsorship, buy-in of the management)*?

How was acceptability among colleagues when you started? So far, is there any improvement *(Probe for belief in authenticity and referrals)*?

What challenges are you yet to overcome and why (It could be personal or as a centre)?

How were colleagues from other departments convinced about using your services?

From your experience, what qualities would classify PC team as a good palliative care team *(probe for qualities to look for in the professionals)*?

What would encourage more people to use our services or create/increase public awareness of our services?

What do you think would hinder people from using these services? *(Probe for ignorance, costs, distance, culture, religion)*

Summary

(Summarize the respondent's answers by itemizing/highlighting crucial points)

Conclusion

Is there something else you would like to communicate about this subject of palliative care at your centre?

Appreciation

(Thank the respondent for their time and sincerity during the interview)
