# Supplementary material for: Barriers and facilitators to the implementation of palliative care services at five tertiary hospitals in Nigeria: a qualitative formative study
Source: BMC Health Serv Res. 2025 Jul 23;25:970. doi: 10.1186/s12913-025-13138-1 (PMC12285048; doi:10.1186/s12913-025-13138-1)
Supplement: Supplementary file 3 — Supplementary Material 3. [file 12913_2025_13138_MOESM3_ESM.docx]

**Additional Quotes on the Implementation of Palliative Care Services in Nigeria**

**Innovation**

Perceived benefits of PC

“The truth is the treatment, the word of encouragement, the workers they talk to us, give us life, give us hope” (Patient, Site A)

“Pain and palliative have really helped us a lot, that at the last moment they came in and kind of performed a miracle. So, in summary, they helped us a lot and they are still helping” (Caregiver, Site E)

“We thank God for palliative care in fact experience in teaching hospital if not because of palliative now with word of advice, courage everything like that, if they don't see us, they will call us to ask about her health, do you know that all these things make patients to get better” (Caregiver, Site C)

**Inner setting**

Informal funding arrangements

“There are some patients that are very rich, they are rich and can afford it and do more. They can say they want to pay 20,000. So that sometimes may cover up for patients that actually don’t have the money to pay.” (Physician, Site B)

Resources and funding support

“Even the one [funding] we use to get from oncology has stopped. We used to, we used to before. Some years back but now they no longer give us. And we don’t have any materials now to work.” (Nurse, Site E)

Organizational structure

“We need office, we need office. This is the only space that we have here. We need office” (Nurse, Site E)

Delayed referral

“They present late as at the point where the patient is gasping for air that is when we are seeing the patient and we have nothing to offer” (Physician, Site B)

**Outer setting**

Traditional care

“With my finding so far over 70% of people with this critical illness are not financially buoyant to take care of it. And by so doing they find a way to get alternative medication and before you know it the thing will give them up and they die.” (Caregiver, Site D)

Quality control of medication

“It is only weak opioid that is available there is no strong opioid” (Nurse, Site C)

**Characteristics of Individuals**

Knowledge of and beliefs about palliative care

“They believe all we do here is break bad news and it’s only those that are ready to die that we take care of, those that are at the later end, the end stage that we take care of, so when they refer patients to palliative care, they will say you’re referred to palliative care, ahh it means you’re going there to die, any patient referred to the palliative care, ahh that’s the end oo” (Nurse, Site A)

Attitudes towards palliative care among non-PC providers

“Even when I see a patient that is supposed to benefit [from palliative care], they are not doing anything about it, I will go and look for the consultant. He will tell me palliative care is dragging his patients with him.” (Physician, Site D)

“Some people, you know, to interact with people to communicate with people, they find it difficult. The skill is not there... And even most of them when they refer patients from the primary team doctors here [at the hospital] or anywhere by the time they are here, they will not want to go back to their primary team doctors because of the services we [palliative care] render. Even when they send patients to us, they will say if it is palliative care, it is the last resort.” (Nurse, Site C)

Patients and caregivers' experiences with non-PC providers

“He didn’t say it professionally. That very day when I did endoscopy because it is of the colon, when they were done, the man just came out and said that it is cancer. At the counter, he just said it and handed me the results. No one to call.” (Patient, Site D)

PC provider attitudes

“The general here at palliative care came and did ward round, so he came to our place that very day, so it was my sister that was ill, then he began to speak to us. I saw joy within him which gave us hope and that she would be okay. He expressed joy toward us. He cares for my sister, he also cares for me, so we saw his love which gave us hope that she will be okay” (Caregiver, Site C)

Financial constraints

“Most of our patients they don’t have anything. Most of them, when they come in the morning for their treatment, they don’t have money, you will feed them from our own personal pocket. Most of them will come without food, the money for doing blood transfusion” (Nurse, Site E)

**Process**

Systematic PC education & training

“For the nurses, they have in-service training that they do. It may not be specifically for palliative care, but I am very sure that they actually mention some aspects of palliative care in the nurses’ in-service training. And for the doctors, most of them do some online courses that may last for like 6 months, 12 months” (Physician, Site B)
